# Conference Report: METROLOGY ISSUES IN TERAHERTZ PHYSICS AND TECHNOLOGY Gaithersburg, MD December 13, 1994 Report prepared by

**DOI:** 10.6028/jres.100.054

**Published:** 1995

**Authors:** Raju Datla, Erich Grossman, Mitchell K. Hobish

**Affiliations:** National Institute of Standards and Technology, Gaithersburg, MD 20899-0001; National Institute of Standards and Technology, Boulder, CO 80303; 5606 Rockspring Road, Baltimore, MD 21209-4318

## 1. Introduction

Tremendous progress has been made in recent years in the development of new sources, detectors, antennas, and materials for the terahertz (THz) spectral region, i.e., from 100 GHz (3 mm wavelength) to 10 THz (30 mm wavelength). Similarly, dramatic improvements have been made in workhorse technologies, such as terahertz Schottky diodes and molecular gas lasers. This progress in device development opens the door to many exciting applications in remote sensing of the Earth’s atmosphere, automotive and military radar, molecular spectroscopy, materials characterization for process control, and astronomy. However, very little attention has been paid to questions of accurate measurement techniques, calibration, and standards at THz frequencies.

In order to discuss metrology issues and the role NIST might play in the rapidly advancing field of THz technology, the staff in various divisions of NIST’s Physics Laboratory, in collaboration with the staff in the Electronics and Electrical Engineering Laboratory (EEEL), convened a Workshop on Metrology Issues in THz Physics and Technology. The workshop was held at NIST on December 13, 1994, and was attended by 50 representatives from industry, academia, and federal agencies.

The speakers for the morning session addressed the general topic of THz applications, with specific reference to applications in remote and atmospheric sensing, commercial applications of THz technology using both coherent and non-coherent sources, and THz applications in microelectronics. The afternoon session’s speakers addressed specific measurement issues, including both coherent and incoherent absolute power measurement, antenna efficiency, spatial beam measurement, and measurement of material properties. Following the afternoon’s presentations, an open discussion addressed the specific areas that NIST should target to support research and development work in the THz region, and the role(s) NIST could play in support of these activities. Many speakers lamented the lack of data on optical properties—emittance, reflectance, and transmittance—of materials in the THz region. Equal concern was expressed at the lack of standards for absolute power measurements in the THz region. *For rapid progress in THz physics and technology the consensus and recommendation was that NIST should address: (1) the measurement, validation, and dissemination of optical properties of materials; and (2) the development of standards for absolute power measurements from both single mode and multimode sources.*

Complete proceedings of this workshop that include the talks presented, details on the program, and a list of attendees can be found in NIST IR 5701, which will be published in the near future.

## 2. Opening Remarks

After an initial welcome by Raju Datla, of the Infrared Radiometry Group at NIST (Gaithersburg), Erich Grossman, Cryoelectronic Metrology Group, NIST (Boulder) discussed prior NIST work at THz frequencies. This work has addressed frequency-related measurements, detector development, and lower frequency (but still THz-capable) techniques. Of particular note was the work done in 1972 by Evenson et al. determining the speed-of-light by measuring both the frequency and the wavelength of the methane stabilized HeNe laser line at ~3.4 μm (88 THz) with a relative standard uncertainty of about 1×10^−9^. For the frequency measurement they had to build a frequency synthesis chain starting from the existing frequency standard, which is the cesium atomic clock at ~10 GHz, all the way to the HeNe line at 88 THz. This measurement was performed with high speed, high bandwidth, nonlinear metal-insulator-metal (MIM) diodes, which were used extensively for harmonic generation and heterodyne mixing. These diodes were largely developed at NIST/Boulder. As a result of the accuracy achieved in these measurements, metrologists decided to change the definition for the meter.

Other techniques and instrumentation developed by NIST include the laser magnetic resonance (LMR) spectrometer, and the tuned far infrared (TuFIR) spectrometer, which were used to measure the transition frequencies of lines from atomic, molecular, and ionic species. Recent work by NIST in this area has been to measure arbitrary lines in the visible spectrum that are separated by THz frequencies from very accurately known visible lines. GaAs Schottky photodiodes are being used as optical photomixers and THz harmonic generators. Also, Josephson junctions with THz characteristic frequencies have been developed, which are useful as heterodyne mixers and harmonic generators when placed at antenna feed points, and as voltage-tunable GHz and THz oscillators when deployed as phase-locked, two-dimensional arrays.

In the area of power measurements, emphasis was placed on development of low-noise, cryogenic detectors. The high *T*_c_ transition edge bolometer developed at NIST/Boulder measured a noise-equivalent power (NEP) of 9 $\text{pW}\sqrt{\text{Hz}}$ which, until recently, was a world record for this kind of measurement.

Grossman concluded with a discussion of THz metrology issues that he hoped would prompt discussion throughout the workshop. These included:

- absolute power measurements, especially for calibration of calorimeters;
- spatial measurement to determine beam quality and to characterize antennas;
- frequency-related measurements for reference frequencies and frequency synthesis, and to determine spectral purity;
- optical measurements to determine optical constants for bulk materials;
- the emittance/reflectance (diffuse and specular) of surfaces; and,
- performance measurements on devices.

## 3. Session I: Applications Overviews

The morning session was chaired by Charles Clark, Chief, Electron and Optical Physics Division, NIST (Gaithersburg), who introduced Peter Siegel, from the Jet Propulsion Laboratory. Siegel addressed the use of THz techniques and devices in remote Earth-sensing applications in his presentation on *Heterodyne Radiometry for Millimeter and Submillimeter-Wave Earth Remote Sensing*. This work, prepared in cooperation with Joe Waters, described the science drivers for microwave remote sensing of the Earth, with specific reference to NASA’s Upper Atmosphere Research Satellite (UARS). This satellite, launched in 1991, has provided valuable data on the distribution of gaseous constituents of our atmosphere, using an instrument called the Microwave Limb Sounder (MLS). The MLS has been used to address details of stratospheric ozone chemistry, upper tropospheric and stratospheric greenhouse gases, measurement of volcanic pollutants in the lower stratosphere, and measurements through ice clouds and aerosols. A general introduction to limb sounding demonstrated the utility of the technique to measure atmospheric constituents. One of the advantages of this technique is that one can measure the vertical distribution of the species. In contrast, ground based column measurements give only total levels, but not their vertical distribution. Heterodyne limb sounding also allows high spectral resolution, has excellent sensitivity, provides simultaneous measurements in many channels, and allows the use of long-life millimeter wave detectors that operate at room temperature.

Siegel addressed the receiver requirements for Earth remote sensing, and noted ways in which they differ from requirements for astrophysical measurements. Generally, the constraints on Earth remote sensing are not as stringent as those for astrophysics, as the signals are stronger and the spectra of Earth’s atmospheric constituents are well known. There are constraints, however: (1) the observation time window is on the order of 1s, as the satellite is moving very quickly in its orbit; (2) there is a limit on power consumption, so the number of radiometers needed to cover spectra of different frequencies cannot be arbitrarily large, and all radiometers that are used must be turned on at the same time to record the spectra of different species simultaneously; and, (3) absolute calibration of the radiometers must be performed. However, the implementation of suitable technologies in the UARS-MLS resulted in data acquisition within 2 days of its deployment, with continuous data having been received for over 39 months.

Siegel then addressed the follow-on to UARS-MLS, the Earth Observing System (EOS) MLS, scheduled for flight as part of NASA’s EOS mission, with specific attention paid to the differences in technology between the two instruments, and the measurement benefits that would accrue. He enumerated the need for data and standards for the scientific experiments planned in the new mission. For radiance calculations some of the needs are: (1) high accuracy emissivity data for materials used in the calibration loads for the frequency range of 100 GHz to 2.5 THz; (2) absolute filter transmittance data; (3) absolute radiometric sideband calibration; and, (4) antenna beam patterns across all RF bands up to 2.5 THz. For instrument calibrations, some of the needs at submillimeter wavelengths are: (1) standards for absolute power measurements; (2) standards for absolute frequency measurements; (3) characterized blackbody-emissivity equivalent-load temperatures; (4) standards for spot noise temperature measurements; and, (5) relative sideband response measurements.

The use of THz techniques in remote sensing was further explored by Kelly Chance, Harvard-Smithsonian Center for Astrophysics, who described THz Applications in Atmospheric Sensing. Most of his work has been on stratospheric sensing of profiles of trace gases involved in ozone chemistry as a function of altitude, based on balloon-borne, Fourier transform spectrometry. These techniques give unique measurements of OH, HO_2_, H_2_O_2_, and favorable measurements of HOCl, ClO, HCl, and HF. These emission measurements yield diurnal behavior. These methods are particularly suitable for stratospheric measurements, where as tropospheric lines are too broad and too obscured by H_2_O to be measured by THz techniques, except in special cases. Chance described the Smithsonian Astrophysical Observatory FIRS-2 instrument, which uses a double-beam Fourier transform spectrometer (FTS), covering the ranges 80^−1^ cm to 210 cm^−1^ to (2.4 THz to 6.2 THz) and 350 cm^−1^ to 700 cm^−1^ with 0.004 cm^−1^ resolution. The fine rotational and vibrational spectral patterns of several species are collected in the Smithsonian Astrophysical Observatory Database, which is available via file transfer protocol (ftp). Given the role(s) of OH in atmospheric chemistry, Chance and his colleagues are developing the OH Interferometer Observations (OHIO) concept for satellite-based measurements of stratospheric OH. This is an option for the generalized far infrared (FIR) Fabry-Perot instrument, optimized for satellite use, and is a collaborative effort of the Smithsonian Astrophysical Observatory, the National Air and Space Museum, the Naval Research Laboratory, and with scientists in The Netherlands and Germany.

The application of THz techniques to characterization of electronic semiconductor materials was described by Larry Carr of the R&D Center/Electronic Materials Laboratory, Grumman Aerospace and Electronics. His presentation on *Applications of Synchrotron Far-IR to Studies of Electronic Materials* described the properties of FIR beams from synchrotrons, and applications. Carr first described the basics of how synchrotron radiation is generated, and then went on to address the features of such radiation. Key to its use is its continuous spectral coverage from microwaves through x rays; that it is two to three orders of magnitude brighter than thermal sources; that it is pulsed (due to electron bunching); and that it is spatially coherent, with small beam divergence. The electrons in a synchrotron storage ring are made to travel in bunches because of the location of an rf cavity in one part of the ring, which provides energy to the electrons to make up for losses, and to prolong their lifetime in orbit. The radiation exits the port as pulses with duration and separation based on the spatial extent of the electron bunch and the separation between bunches. In the case of a single bunch, the pulse repeats each revolution. The radiation exits as a highly collimated beam with a small divergence angle because of the relativistic speeds of the electrons. This latter feature is very useful for IR microspectroscopy. The pulse characteristic is used to determine response times for high-speed FIR detectors such as quantum well infrared photodiodes (QWIP) and high *T*_c_ superconductors. As an example, Carr showed results of a study of the responsivity of an AT&T quantum well infrared detector, with a measured response time of less than 500 ps. The IR radiation pulse has also been used as a probe for pump-probe spectroscopy of detector materials, such as GaAs and HgCdTe. Examples of this work were shown for undoped GaAs, which revealed the scattering rates and time dependence for electrons and holes.

Material characterization was also addressed by Jim Allen, from the Center for Free-Electron Laser (FEL) Studies, University of California, Santa Barbara (UCSB), who spoke on the *Applications of Free-Electron Lasers as Terahertz Sources*. Allen described some details of the FEL facilities at UCSB, and characteristics of the radiation they produce: The radiation is tunable over the range 120 GHz to 4.8 THz; it is quasicw, in 1 μs to 20 μs pulses with a fractional frequency instability of approximately 10^−6^; its power output is from 500 W to 5 kW. These characteristics make such radiation ideal for examining materials properties in the gap between electronic and photonic regions, and enable its use to examine nonlinear quantum transport in semiconductor nanostructures. Allen described two applications: definition of high frequency limits in resonant tunneling diodes, and photon-assisted tunneling in semiconductor structures. The potential impact of this technique on technology will be to define the high frequency limits of conventional electronics and to enable new electronics at THz frequencies.

A fascinating new concept in THz beam generation was described by Daniel Grischkowsky, Oklahoma State University, School of Electrical and Computer Engineering, in his presentation on *Femtosecond THz Beam Generation and Applications*. This optoelectronic approach uses a charged coplanar transmission line circuit irradiated by a laser. With a potential across the gap the line generates a current pulse when hit by a laser pulse at the gap. The current pulse drives a dipole antenna and radiates at THz frequencies. The semiconductor is fabricated to give a response of less than 1 ps. The same principles are used with a similar semiconductor chip to detect the THz radiation. This technique can give a THz signal-to-noise ratio (SNR) of 1000:1. Grischkowsky showed the results of an optimized optoelectronic system radiating THz radiation with pulse widths on the order of femtoseconds, with an SNR on the order of 10 000:1. Perfect synchronization between the transmitter and receiver is possible. The combination of an ultrafast source and detector can then be used to do time-domain spectroscopy to examine the dynamics of several gas species, e.g., N_2_O, H_2_O, to perform non-contact characterization of materials such as n- and p-type GaAs, to perform ranging measurements, and to examine temperature distributions in flames.

## 4. Session II: Specific Measurement Areas

Having discussed several application areas, the Workshop next focussed on metrology issues in a session chaired by Kenneth Evenson, Time and Frequency Division, NIST Boulder. Evenson introduced Neil Erickson, from Millitech, who gave a presentation on *Power Standards for the Near Millimeter and Submm Region*. In an incisive talk, Erickson described the state of the art with respect to power standards in the GHz range, stating that standards are poorly established above 90 GHz, and nearly completely absent above 140 GHz, to the point where the manufacturers of devices cannot state with complete certainty the accuracy and precision of their instruments in these regions. This lack of standardization makes it difficult to verify theories or to confirm expectations, leading to uncertainties in local oscillator power for mixers, efficiencies of multipliers, laser output power, and more. Erickson went on to describe power measurement problems in the submillimeter region, where available power is small from most sources, and accurate calorimeters are not sufficiently sensitive. Unknown harmonic content, spurious oscillations, and contaminating lines make it difficult to verify purity. Erickson described the advantages and disadvantages of several types of power sensors including waveguide-mounted sensors (waveguide calorimeters, waveguide thermistors or thermocouples, and diode detectors), and quasi-optical devices, such as quasi-optical laser power meters (thermopiles), acoustic wave sensors, pyroelectric sensors, and thin-film bolometers, all of which have possible nonuniform absorption characteristics. Based on these deficiencies, Erickson described the parameters necessary for an improved waveguide calorimeter design, which could be an excellent general-purpose sensor throughout the submillimeter region if speed and drift could be improved. It requires two matched sensors, as it is a differential measurement device; the lower limit to measurable temperature is set by the thermal isolation of the elements and the match in the drift of the two sensors. It should be feasible to achieve a 10 μW measurement level and a time constant of less than 10 s with an uncertainty of a few percent. He described a prototype device that he has constructed based on a WR-10 waveguide input, intended for use between 80 GHz to 1 THz. It has a responsivity of 100 K/W and a time constant of 7 s. The root means square drift is 7 μW, which could be improved by better insulation. He expressed the view that NIST involvement could help produce a better device.

An excellent example of the need for well-characterized, standardized, and calibrated measurement tools was provided by John Mather, NASA Goddard Space Flight Center, who described the important results obtained with the *Far Infrared Absolute Spectrophotometer (FIRAS)* instrument, flown on the Cosmic Background Explorer (COBE) satellite. The purpose of this measurement was to compare the cosmic microwave background to an accurate blackbody in an effort to provide information about the earliest epochs in the history of the observable universe as a test of the Big Bang Theory of cosmology. FIRAS is intrinsically a differential device, matching observation against a perfect blackbody. This ordinary differential interferometer has two inputs and two outputs, which are used to generate an interferogram. The blackbody is isothermal (to within 1 mK), operating at liquid He temperatures (1.5 K), and can be heated to change the temperature of the device over the range from 2 K to 20 K. The detector is a bolometer, using a diamond substrate blackened with chromium gold alloy, and a heavily doped silicon chip thermister. Painstaking calibration produced measurements that demonstrate that there is no deviation between the temperature of the observed cosmos and that of a blackbody curve at 2.726 K; there was no deviation found at <0.03 % of peak from 0.5 mm to 5 mm, thereby confirming the Big Bang Theory.

Astrophysical measurements provide one of several motivations for *Laboratory Spectroscopy in the THz Region*, as discussed by Geoff Blake, California Institute of Technology. By exploring this region with a widely tunable system, Blake and his coworkers are able to obtain high spectral resolution and sensitivity near the quantum limit. This provides a tool for rotational spectroscopy for astronomy and atmospheric remote sensing. Linewidth requirements are very stringent in these applications. Heterodyne receivers up to the 1.5 THz region are needed to cover *ortho*- and *para*- H_2_O lines. Similarly, in the laboratory, THz measurements are needed to examine diatomic molecules by measuring rotational and vibrational eigenstates to get potentials. These parameters are necessary to fully understand molecular interactions in chemistry and biochemistry, where bonds vibrate in the mid-IR (400 cm^−1^ to 4000 cm^−1^). FIR measurements would allow characterization of weaker bonds or heavier molecules, such as found within and between proteins. Such interactions are key to the pharmaceutical industry, where quantitative structure modeling is used in the rational design of new drugs. Blake spoke about laboratory spectroscopy measurements on molecules using FIR lasers modulated by microwaves using semiconductor mixers. New applications are becoming available using heterodyne sources obtained by photomixing of optical lasers using optoelectronic devices built with GaAs to provide pulse widths on the order of 150 fs. Several such devices have been built, and will be flown on the Kuiper Airborne Observatory in April 1995 to do submillimeter astronomy, looking for ground-state ^18^O ^16^O isotomer of the oxygen molecule in natural abundance at near-THz frequencies. In addition, a miniaturized device is being designed to fly on the Perseus remotely piloted vehicle and ER2 platforms to take stratospheric measurements on small scales, on the order of 100 m, on the order of cloud sizes. These optical photomixers are broadband, have no mechanical tuners over the full bandwidth range from DC to 1.5 THz, and, in principle, can be interfaced to computers to allow complete control of instrument operation and data acquisition.

A similar THz source has been developed by Alan Pine and Richard Suenram of the Molecular Physics Division at NIST Gaithersburg, in collaboration with Elliott Brown and coworkers at MIT Lincoln Laboratory who have fabricated a new, low-temperature-grown, GaAs device that acts as an ultrafast photomixer. Pine has incorporated this device into a broad-band THz spectrometer using two narrow-linewidth (1 MHz) dye lasers. The spectrometer operates by focusing the output of the two dye lasers onto the ultrafast photomixer; the output of the photomixer is the difference frequency between the two dye lasers. Holding one dye laser frequency fixed, and tuning the frequency of the second, results in broad-band, tunable output in the far infrared region. The output is passed through an absorption cell containing the compound of interest, and the radiation is detected by a helium-cooled bolometer. This spectrometer has been used to obtain high-resolution spectral data from 150 GHz to 1 THz; Lincoln Laboratory has fabricated newer devices that should operate to 4 THz. In support of this work, NIST is working on implementing solid-state diode lasers as pump sources for the ultrafast photomixers. Once the linewidths of the diode laser sources have been reduced to <1 MHz and suitable locking schemes developed, computer control to make the new instrument “user-friendly” will be implemented.

Extending the utility of THz measurements into the realm of materials science and technology, Robert Giles, from the Submillimeter Technology Laboratory, University of Massachusetts, Lowell, described the *Characterization of Material Properties at Terahertz Frequencies*. The main goal of this work is to acquire millimeter wave radar cross-section signatures of hard bodies, such as tanks in a battlefield, using submillimeter-wave model measurements. This requires the knowledge of optical properties of materials at submillimeter wavelengths in order to develop realistic models for scaling, etc. To support these goals, they must produce high-fidelity, scale replicas of complex metallic structures, design a wide range of optical properties measurement systems using current submillimeter wave source/detector technology, establish precise calibration standards, and scale millimeter wave dielectric properties of composite materials at submillimeter wave frequencies. Four techniques have been explored: submillimeter ellipsometric measurement, high-precision submillimeter wave reflectometry, laser-based Brewster’s angle measurements, and FIR Fourier transform spectroscopy. Giles showed details of design and development of suitable instrumentation, and the use of these techniques in their laboratory specifically for evaluating the optical properties of materials at THz frequencies. They have established calibration standards for performing reflectivity measurements to a repentability of ±0.1 %. Also, they have developed a variety of artificial dielectric materials for bulk and thin film applications, and have tailored their optical properties for the fabrication of frequency-selective absorbing structures.

Continuing in the vein of calibration-related activities, Gabriel M. Rebeiz, Electrical Engineering/Computer Science Department, University of Michigan, Ann Arbor, described his work in the area of *Planar Antennas, Power Meters, and Calibration Techniques at Terahertz Frequencies*. Rebeiz showed some photomicrographs of beautifully etched antennas, clearly demonstrating the state of the art in design and fabrication of planar antennas. He showed their normalized antenna patterns and compared them with theoretical predictions. In most cases there was good quantitative fit; even where experimental values differed from theory, the qualitative fit was excellent. His group’s designs for reflector antennas integrated with detectors show extremely high gain (>30 dB) with very small size and good coupling efficiency (84 %) to Gaussian beams, making them suitable for radiometric and communications applications. Using similar principles, they have been able to construct easy-to-build, monolithic THz power meters that are accurate to within ±5 %, and can be arrayed for spatial sampling. Rebeiz went on to describe several methods to calibrate antenna gain. The two-antenna method, the radiometric method, and the plane wave method were discussed. He showed the areas of applicability and problems associated with each method.

Harold Fetterman, Electrical Engineering Department, University of California, Los Angeles, addressed issues of *Optoelectronic Measurement Techniques*. He described measurements performed in his laboratory using picosecond lasers and optical switches. His group was able to measure the current gain and the optical response of an AlInAs/GaInAs high electron mobility transistor (HEMT), and similar characteristics of an AlGaAs/GaAs heterojunction bipolar transistor (HBT) up to 100 GHz. He concluded that for frequencies above 100 GHz, no measurement techniques are available. Fetterman expressed the view that NIST should address measurement techniques for high frequencies and develop standards for, e.g., testing repeatability. Several electro-optical sampling techniques were discussed, including frontside probing of microstrip transmission lines, and backside probing of coplanar transmission lines. Coplanar photoconductive switches have been designed and implemented, as has a pulsed millimeter wave radiation experiment, used to measure radiation from 45 GHz to 75 GHz in real time using heterodyne detection. Using microfabrication techniques, Fetterman and his group have been able to design repetition rate multipliers, power splitters, and optical delay lines suitable for use in optoelectronic applications, as well as integrated optical waveguide-HBT structures, which they have characterized. Fetterman expressed the view that it would be a real breakthrough to put THz signals on optical signals and use the enormous bandwidth that is available, for example 2000 GHz at 1.3 μm, for communications. Such implementation is a real possibility, as he outlined how optical techniques could be used to generate, detect, and transmit THz radiation. He recommended that NIST develop standards and techniques which would make THz technology commercially viable for communications.

## 5. Round-Table Discussion

As a lead-in to the round-table discussion, Neil Erickson was asked to provide some information about the market for millimeter and submillimeter components and full systems, based on his company’s data. Major volume applications consist of imaging systems for aircraft landing and contraband detection, and short-range radar for automobile collision avoidance. Smaller markets are expected for submillimeter wave systems. Based on his perspective from the commercial sector, Erickson offered the following as examples of what NIST could offer industry:

- calibration of power from sources;
- characterization of absorbers for radiometer calibration and antenna measurements;
- waveguide standards at frequencies above 325 GHz; and
- quasi-optics and antennas, standard gain horns, and low-gain probes for near-field scanning.

Using these suggestions as a jumping-off point, the Discussions chairman, Richard Harris, Chief, Cryoelectronic Metrology Division, NIST/Boulder, opened the floor for discussion by requesting that members of the three major participating groups—industry, academia, and government—tabulate what they each felt were suitable areas for NIST to address. Much discussion ensued, with key points recorded for later analysis. Topics raised included:

- THz modulation of optical communication;
- FTIR systems;
- industrial applications, including imaging for aircraft landing, volcanic SO_2_, collision avoidance, and contraband detection;
- frequency measurements and phase noise;
- tools for high frequency measurements, (e.g., sampling, vector network analyzers);
- calibration round-robins;
- spatial measurements of antenna characteristics;
- provision of standard reference materials;
- source comparisons;
- standard attenuators;
- environmental monitoring;
- phased arrays for mapping;
- generation of standards for absolute power;
- calibrated sources;
- optical properties of materials; and
- instruments to support process monitoring in several industries.

After discussion of the relative merits of each of these suggestions, and some extended debate over NIST’s proper role with specific reference to its charter, a vote was taken to determine which of the above areas should most properly be addressed by NIST. The attendees at the workshop (excluding NIST staff) were asked to vote. The most votes (in descending order) were for NIST to:

- take an active role in the measurement, evaluation, and dissemination of optical properties of materials (16);
- provide absolute power standards (9 for single mode, 6 for multimode);
- provide tools for frequency measurements (5);
- provide standard reference materials (4); and
- provide calibrated sources (4).

Several other topics received less than four votes. NIST’s participation in collaborative efforts was discussed, as was its potential role in forming the nucleus of such efforts.

The attendees expressed great pleasure in the format and outcome of the workshop, and look forward to further meetings of this sort as industry, academia, and government organizations move further into the THz realm.

